# The Importance of Reaction Conditions on the Chemical Structure of *N*,*O*-Acylated Chitosan Derivatives

**DOI:** 10.3390/molecules24173047

**Published:** 2019-08-22

**Authors:** Agnieszka Piegat, Agata Goszczyńska, Tomasz Idzik, Agata Niemczyk

**Affiliations:** 1 West Pomeranian University of Technology Szczecin, Faculty of Chemical Technology and Engineering, Polymer Institute, Division of Functional Materials and Biomaterials, 45 Piastow Ave, 70-311 Szczecin, Poland; 2 West Pomeranian University of Technology Szczecin, Faculty of Chemical Technology and Engineering, Department of Organic and Physical Chemistry, 42 Piastow Ave, 71-065 Szczecin, Poland

**Keywords:** acylation reaction, chitosan derivatives, EDC/NHS coupling system, fatty acids, spectroscopic methods

## Abstract

The structure of acylated chitosan derivatives strongly determines the properties of obtained products, influencing their hydrodynamic properties and thereby their solubility or self-assembly susceptibility. In the present work, the significance of slight changes in acylation conditions on the structure and properties of the products is discussed. A series of chitosan-acylated derivatives was synthesized by varying reaction conditions in a two-step process. As reaction media, two diluted acid solutions—i.e., acetic acid and hydrochloric acid)—and two coupling systems—i.e., 1-ethyl-3-(3-dimethyl-aminopropyl)-1-carbodiimide hydrochloride (EDC) and *N*–hydroxysulfosuccinimide (EDC/NHS)—were used. The chemical structure of the derivatives was studied in detail by means of two spectroscopic methods, namely infrared and nuclear magnetic resonance spectroscopy, in order to analyze the preference of the systems towards *N*- or *O*-acylation reactions, depending on the synthesis conditions used. The results obtained from advanced ^1^H-^13^C HMQC spectra emphasized the challenge of achieving a selective acylation reaction path. Additionally, the study of the molecular weight and solution behavior of the derivatives revealed that even slight changes in their chemical structure have an important influence on their final properties. Therefore, an exact knowledge of the obtained structure of derivatives is essential to achieve reaction reproducibility and to target the application.

## 1. Introduction

Chitosan is a random copolymer, containing (1→4) linked 2-acetamide-2-deoxy-β-d-glucopyranose and 2-amino-2-deoxy-β-d-glucopyranose residues. The presence of two functional groups, amino and hydroxyl, in the chitosan structure strongly determines its unique properties and enables various physical and chemical modifications to create derivatives with widening applications—e.g., in the biomedical field [1,2], in water treatment [3] or as antifungal and antioxidant materials [4]. Therefore, many efforts have been made to chitosan chemical modification in order to obtain derivatives with improved solubility, photocrosslinkability, biocompatibility, antibacterial activity or other advanced properties [5].

As a consequence of amino groups’ presence in macromolecular chains (defined by the degree of deacetylation), chitosan exhibits polycationic characteristics in acidic aqueous solutions. The protonation of amino groups strongly influences the conformation of macromolecules in a solution and could be controlled by changing the pH or the ionic strength of the solution. The behavior of chitosan chains in diluted acids has already been investigated and it is well known that a higher ionic strength or lower pH result in a higher protonation of amino groups and under such conditions, macromolecules behave more like rigid rods [6,7]. As a consequence of this specific arrangement of the chains, the viscoelastic properties of the chitosan solutions strongly depend on the polymer concentration [8] since the entanglement effect for the rod-like polymers occurs at a significantly lower concentration than for flexible polymers. This fact is significant from a chemical point of view, as it also affects the accessibility of the functional groups during a chemical reaction. Additionally, the behavior of chitosan once protonated can be advantageous due to several practical features: it has a tendency to create polyelectrolytes complexes (e.g., with negatively charged sodium alginate) [9,10], it easily complexes with heavy metals [11,12] and offers improved selectivity for its physical and chemical modification [13].

In this regard and considering the different reactivity of the two main functional groups present in chitosan structure, –NH_2_ and –OH, numerous derivatives have been reported through selective acylation of the amino group (*N*-acylation) [14,15,16]. In contrast to *N*-acyl chitosan, derivatives modified only at the –OH positions are exceptional, and most of the attempts found in the literature for selective *O*-acylation are rather complex and need several protection/deprotection steps, and even these can result in a mixture of *N*-acylated and *O*-acylated derivatives [17,18]. There are few studies in the literature which achieve a selective *O*-acylation of hydroxyl groups with a high yield by carrying out a simple procedure. In these works, a higher level of *O*-acyl chitosan than N-acyl chitosan was achieved in the presence of strong acidic residues, with ${\mathrm{HSO}}_{4}^{-}$ [19] or ${\mathrm{CH}}_{3}{\mathrm{SO}}_{3}^{-}$ acting as counterions [20]. In this sense, the ionic protection of the amino group seems to be a good direction for establishing an alternative to chitosan *O*-acylation.

Concerning the most performed chitosan *N*-acylation modification, common procedures involve acyl halides and anhydrides to introduce the amide group into the chitosan backbone. Additionally, it is often accepted that the use of carboxylic acids, being much less reactive compounds, will allow for a better control of the reaction, which can be advantageous as it imparts a higher selectivity towards the reaction. When a carboxylic group is used for amide formation reactions, its activation by means of creating more active ester using a coupling agent is necessary [21]. The most commonly used is 1-ethyl-3-(3-dimethyl-aminopropyl)-1-carbodiimide hydrochloride (EDC·HCl, abbreviated as EDC), used alone or with an additive to improve the reaction efficiency, usually *N*-hydroxysuccinimide (NHS) [22,23]. Most of the literature concerned with the *N*-acylation of chitosan by employing the different procedures of EDC or EDC/NHS activation recognizes them as quick and facile methods to obtain diverse chitosan derivatives, such as chitosan containing fatty acids [24], deoxycholic acid [25] or methoxy poly(ethylene)glycol (mPEG) [26]. However, in those studies, no protection steps were performed and only *N*-acylation was discussed, assuming that only amino groups, as a stronger nucleophile, underwent the reaction. Nevertheless, when the hydroxyl groups are also present in the structure, as in chitosan or proteins, *O*-acylation can occur in parallel and suppositions about sole amide formation can be misleading [27,28].

Due to the significant influence that the final structure of the acylated chitosan derivatives can have over the final properties of the obtained products, in the present work, the significance of slight changes in acylation conditions employing EDC and EDC/NHS coupling systems to introduce fatty acid moieties into the polymer structure will be discussed. Common reaction conditions will be applied, two acids—acetic acid, and hydrochloric acid—will be studied, and different pH conditions, and chemical structures of the chitosan derivatives obtained will be specifically correlated to the reaction parameters. The ultimate aim is therefore to shed light on the practical meaning of the acetylation degree and path (*N*- vs. *O*-acylation) and to demonstrate the importance of choosing the proper coupling agent and reaction conditions to obtain the desired results.

## 2. Results and Discussion

### 2.1. Synthesis and Characterisation of Chitosan Derivatives—Effect of Reaction Conditions over Acylation Type (N–, O–), Substitution Degree and Chemical Structure

**Scheme 1:**
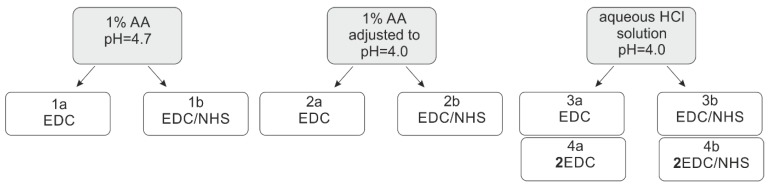
Reaction conditions applied for chitosan derivative synthesis.

Chitosan derivatives (1a–4a, 1b–4b) were obtained by the chitosan acylation reaction using linoleic acid as an acylating agent. Reactions were conducted in three different reaction media—i.e., 1 wt% acetic acid (AA) (1a, 1b; pH = 4.7), 1 wt% AA adjusted using HCl (2a, 2b; pH = 4.0) and aqueous HCl solution (3a, 3b, 4a, 4b; pH = 4.0), with EDC or EDC/NHS (Scheme 1).

**Scheme 2:**
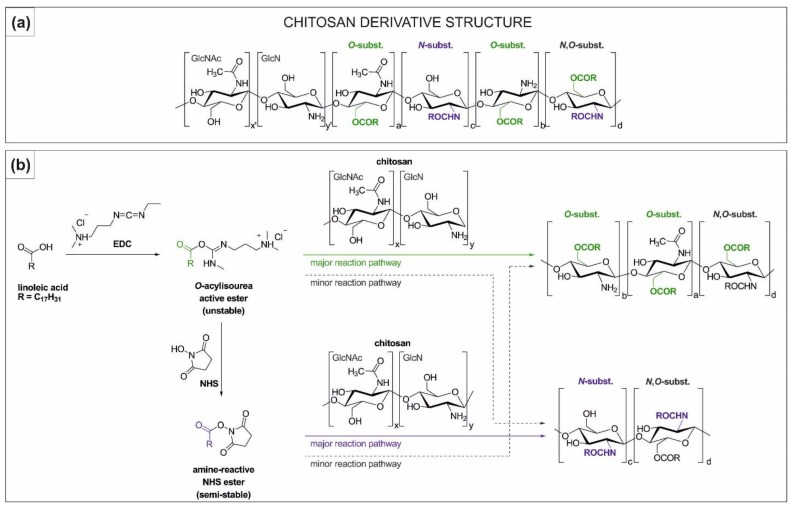
Chemical structure of (**a**) chitosan derivatives and (**b**) a possible reaction pathway.

Due to the presence of two nucleophilic groups in the chitosan structure, there are two possible acylation pathways by *N*- and *O*-acylation (Scheme 2a). The protonation degree of chitosan amino groups can vary as a consequence of pH and acid strength changes, and although there is a general shortage of awareness in the literature about this fact, the importance of the investigation on various reaction media should not be overlooked. Therefore, the final derivative structure could be altered due to the protonated amino groups becoming less reactive and as a result, *O*-acylation could predominate over *N*-acylation.

In the acylation reaction, the activation of carboxylic groups is required due to the weak acylating properties of the carboxylic acids, and carbodiimides, such as EDC, are commonly used for this purpose [29]. The active ester intermediate (*O*-acylisourea ester) created with EDC is able to react with any nucleophilic groups, amino and hydroxyl (possible *N*,*O*-acylation; Scheme 2b) that are present in the chitosan structure, which is unfortunately omitted in some research related to the chitosan acylation using similar conditions to those employed here [30,31].

Due to the susceptibility to hydrolysis of the reactive *O*-acylisourea, typically *N*-hydroxysulfosuccinimide (NHS) is used as an additive in order to convert it into a more stable NHS-ester. Despite the fact that NHS-ester can selectively react with the amino group and, therefore, the *N*-acylation of chitosan should become predominant (Scheme 2b, major reaction pathway), the acidic environment and thus the amino groups’ protonation might still hinder the NHS-ester reaction.

#### 2.1.1. FTIR Analysis

**Figure 1:**
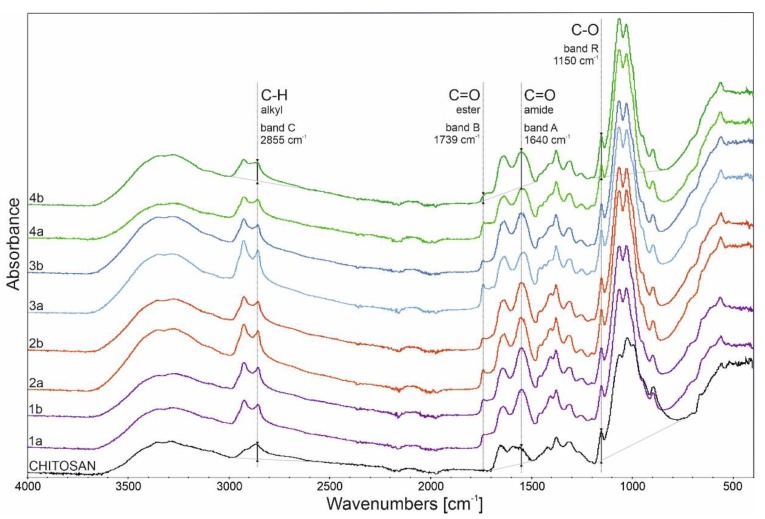
Infrared spectra of chitosan and chitosan derivatives.

The competitiveness of *N*- and *O*-acylation was assessed based on the IR spectra analysis of the derivatives. Figure 1 shows an increase in the intensity or appearance of new absorption bands, confirming the occurrence of simultaneous *N*,*O*-acylation for all the derivatives. The observed characteristic bands of interest are: alkyl C–H stretching and bending in the range of 3005–2840 cm^−1^ and at 1435 cm^−1^; amide I C=O stretching at 1640 cm^−1^ being indicative of *N*-acylation; ester C–O stretching at 1250 cm^−1^, and 1198 cm^−1^, and ester C=O stretching at 1739 cm^−1^ being indicative of *O*-acylation. As can be observed, depending on the reaction conditions as well as the coupling system employed, the characteristic bands vary in their intensity.

**Figure 2:**
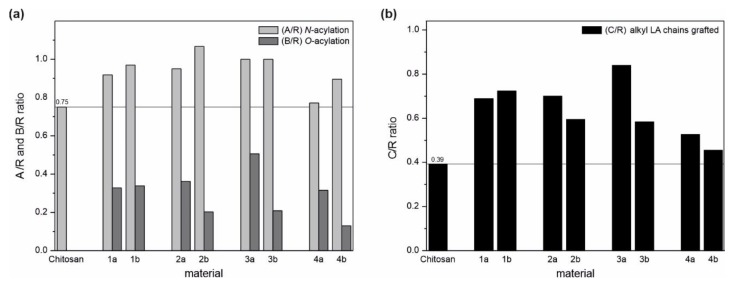
Relative amount of (**a**) amide (*N*-acylation), ester (*O*-acylation) and (**b**) alkyl groups represented by bands ratio.

Analysis of the linoleic acid substitution was done by the band intensity comparison method. The relative number of the particular functional group was obtained by dividing the characteristic band intensity (A- amide, 1640 cm^−1^; B- ester, 1739 cm^−1^; and C- alkyl, 2855 cm^−1^) by the reference one (R, 1150 cm^−1^). The relative number of the amide group (A/R ratio) for chitosan was 0.75 (due to the initial *N*-acetylation of the polymer) and the observed increase in the ratio indicated that all derivatives underwent an *N*-acylation reaction (Figure 2a), with varying ratios from 0.77 to 1.07, which can be correlated with the synthesis conditions.

Chitosan derivatives were prepared with different solvent and coupling agent systems as shown in Scheme 1. In general, by using the EDC/NHS coupling system derivatives with a higher degree of *N*-substitution (i.e., a higher selectivity towards *N*-acylation over *O*-acylation; Scheme 2b, major reaction pathway) were obtained (derivatives 1b–4b), when compared with the derivatives synthesized with EDC alone under the same conditions (Scheme 2b, major reaction pathway). This fact is in agreement with the literature data, where it is pointed out that the NHS-ester promotes amidation reaction [32,33]. Additionally, lower pH values seem to be more likely to occur during *N*-acylation compared to *O*-acylation, when using the EDC/NHS system and AA as a reaction medium (derivative 2b over derivative 2a). Findings relating to the solvent influence show that AA media promote higher *N*-acylation than HCl when EDC and NHS are present in equimolar amounts at pH4. At a given molar concentration, HCl can be more ionized than AA in aqueous solution, interacting strongly with the cationic chitosan resulting in a better protection of amino groups and therefore, lowering their reaction rate. The HCl aqueous reaction media with an equimolar amount of EDC (derivative 3a) led to the highest substitution degree of chitosan. The excess of EDC (4a, 4b) resulted in the lowest chitosan substitution degree, regardless of the coupling system used.

Although, by using EDC/NHS coupling, the selectivity of the reaction can be displaced towards *N*-acylation, it is worth noting that all derivatives synthesized with this system exhibited lower substitution degrees (C/R, understood as a relative amount of alkyl LA chains grafted, Figure 2b) compared to derivatives obtained with EDC. When the chitosan molecules are protonated (as in the reaction media employed), it can be surmised that the EDC-active ester is able to react either with amino or hydroxyl groups (Scheme 2b, major and minor reaction pathway) while, as stated before, for the NHS-ester, *O*-acylation is not the preferential reaction path (Scheme 2b, minor reaction pathway). Considering the substitution place and as observed in Figure 2, this can be corroborated as the *O*-acylation reaction (A/R ratio) exceeds the *N*-acylation (B/R ratio) for derivatives synthesized with the EDC coupling system (derivatives 1–4a) in contrast to the ones obtained using EDC/NHS.

The carboxylic acid used for chitosan modification, which has a specific chemical structure and thus properties (such as solubility or pKa) also affects the reaction pathway [34], therefore one should take it into account when determining the reaction conditions toward a desirable derivative.

#### 2.1.2. NMR Analysis

Nuclear magnetic resonance (NMR) spectroscopy analysis was used to evaluate the chemical structure of the derivatives. The application of common solvent signal suppression techniques, namely watergate and water presaturation, affected the intensities of the signals near the water resonance (Supplementary Materials, Figure S1). Therefore, the diffusion-edited NMR experiment (ledbpgp2s1d) was used for the effective suppression of the residual solvent signals while retaining the signals from the analyzed product. The NMR measurements were performed in a mixture of 2:8 (*v*/*v*) 1 wt% *w*/*v* formic acid-d4 in deuterium oxide and methanol-d4 (FA:MeOH) following the dissolution procedure stated in the Materials and Methods section. This solvent system was selected as allowed for the complete dissolution of all the derivatives (SI, Table S2).

Representative ^1^H NMR spectra of linoleic acid, chitosan, and the derivative 2a are shown in Figure 3 (^1^H NMR spectra of all derivatives (1–4) are included in SI, Figures S3–S10).

The appearance of the characteristic linoleic acid signals in the NMR spectra of the derivative confirmed the successful grafting of the fatty acid chain onto the chitosan. The signal at chemical shift (δ) 2.31 ppm is attributed to the alpha methylene group of the linoleic acid chain and it is downfield compared to the LA spectrum. The resonance signals of terminated methyl and methylene protons are located between δ 0.8 and 1.7 ppm. The signal at δ 5.34 ppm is assigned to the protons of the carbon–carbon double bond in LA; its small integration is due to partial hydrogenation of the bond during the reaction and/or purification process. The mono-allylic methylene protons are overlapped by the signal of three protons of the *N*-acetyl group in *N*-acetyl-*D*-glucosamine residue (GlcNAc). The *bis*-allylic group signal is unnoticeable in the spectrum due to its low intensity. The proton resonance at δ 3.10 ppm is assigned to the H2 proton of the chitosan glucosamine (GlcN) unit. The ring protons (H3–H6 of GlcN and H2–H6 of GlcNAc) are located as a set of overlapping signals in the range of δ 3.4 to 4.1 ppm, and the anomeric proton H1 of GlcNAc resonates at δ 4.55 ppm. The visible changes within the pyranose unit signals are related to the substitution of the hydroxyl and/or amino group.

The ^13^C DEPT–135 NMR spectrum of the product 2a is given in Figure 4. Six signals of chitosan macromolecule at δ 99.3 ppm (C1), 78.3 ppm (C4), 76.4 ppm (C5), 71.7 ppm (C3), 61.4 ppm (C6) and 57.4 ppm (C2), are attributed to the glucopyranose carbons of the GlcN and GlcNAc units. The appearance of new signals was observed at δ 168.9 ppm and also in the range δ 36–14 ppm. The first one is assigned to the carbonyl group, and the signals in the high field region come from methyl and methylene carbons in LA moiety. These results are consistent with the ^1^H NMR analysis.

To determine the place of substitution, a ^1^H–^13^C HMQC experiment was performed for the selected derivative 2a (Figure 5) in the ranges 0–100 ppm and 0–5.00 ppm for carbon and proton chemical shifts, respectively. The ^1^H–^13^C correlation of H1/C1 appears at δ 4.87 ppm/δ 97.72 ppm; those for H3–H5/C3–C5 are observed in the ranges δ 3.55–4.0 ppm/δ 70.16–76.75 ppm. The carbon C2 and C6 at δ 55.49 ppm and δ 59.89 ppm show two hydrogen correlations at δ 3.50 ppm and δ 3.12 ppm (H2) and δ 3.76 ppm and δ 3.95 ppm (H6), respectively. The splitting of the signals (C2–H2, C6–H6) into two peaks, is related to partially substituted hydroxyl and amino groups of chitosan, which confirms the *N*,*O*-acylation in agreement with the FTIR results. The ^1^H–^13^C correlation of acyl group is visible at δ 2.05 ppm/21.57 ppm. The remaining cross peaks in the HMQC spectrum originate from linoleic acid.

**Figure 3:**
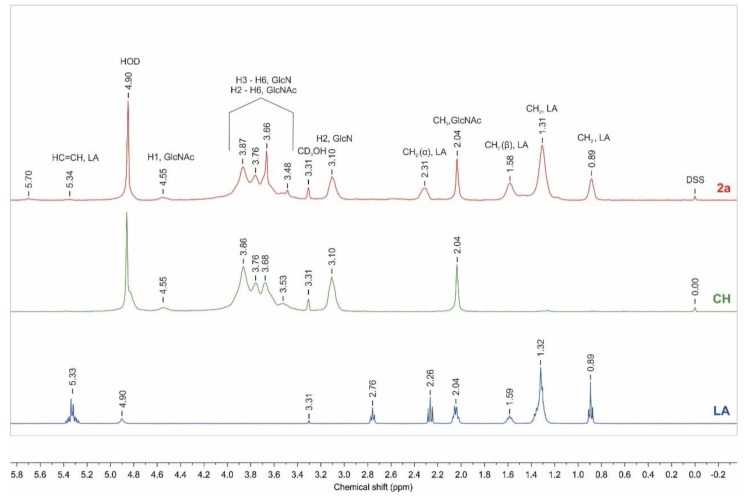
^1^H NMR spectrum of linoleic acid, chitosan and the chitosan derivative 2a.

**Figure 4:**
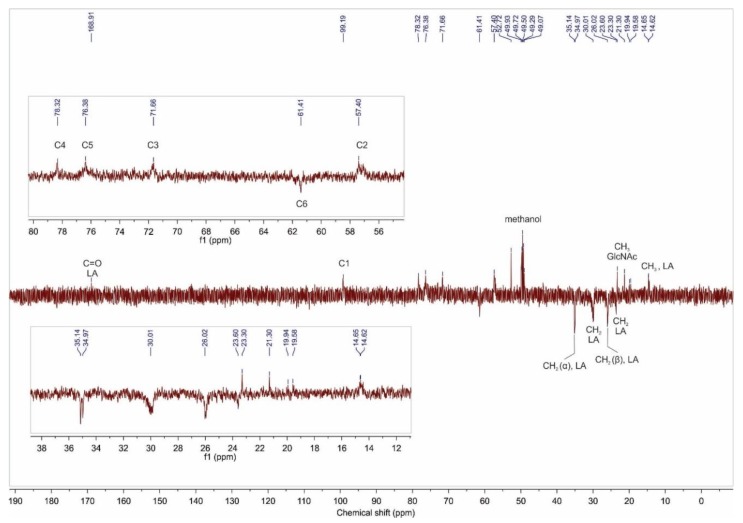
^13^C NMR DEPT-135 spectrum of the chitosan derivative 2a.

**Figure 5:**
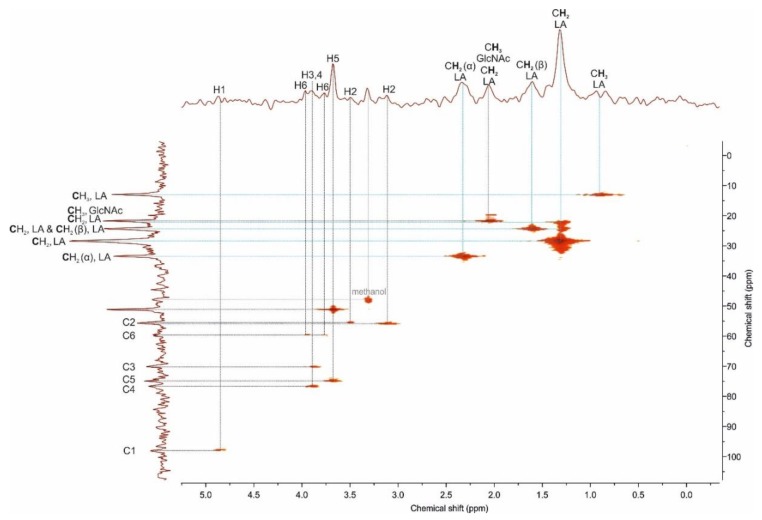
^1^H–^13^C HMQC spectrum of the chitosan derivative 2a.

The compliance of the presented NMR analysis proved the overlapping of CH_2_ α, LA signals concerning an ester or amide group (Figure 3, derivative 2a, δ~2.31 ppm) which leads us to a conclusion that the calculations of substitution degree for *N*- and *O*-acylation can produce incorrect values.

### 2.2. Molecular Weight Determination

The number-average molar weight (M_n_), weight-average molecular weight (M_w_) and polydispersity index (M_w_/M_n_) of the chitosan and its derivatives were determined by gel filtration chromatography (GFC) and the results are presented in Table 1.

The M_n_ and polydispersity of chitosan were around 20 kDa and 7.3, respectively. All the derivatives, except derivative 4b, presented a decrease in M_n_ and M_w_ values. These observed changes in the molecular weight of the derivatives can be related to their specific behavior in acetate buffer. It is well known that acetate buffer, as a nonsolvent for hydrophobic moieties (containing at least 6 carbons in aliphatic chains), promotes some aggregation of amphiphilic macromolecules [35,36]. Those aggregates were probably removed during the filtration process applied before measurement, resulting in an observed decrease of molecular weight. The exception, derivative 4b, exhibited an identical molecular weight to chitosan, showing that a low substitution degree (based on FTIR analysis, Figure 2) did not influence the macromolecules’ behavior in solution. The fact that the derivative 4b did not show a decrease in molecular weight, indicates that the lower M_n_ and M_w_ of the other derivatives is more likely due to their aggregation in solution than to the applied reaction conditions resulting in acidic degradation/depolimerization of the systems.

### 2.3. Behavior in Solution

In terms of future applicability of the obtained systems (i.e., in pharmacy or biotechnology among others), the knowledge of chitosan derivatives conformation and behavior in solution are of key importance. Therefore, solubility, as well as the diffusion coefficients of chitosan and their derivatives were assessed using UV-Vis spectroscopy and dynamic light scattering (DLS), respectively.

**Figure 6:**
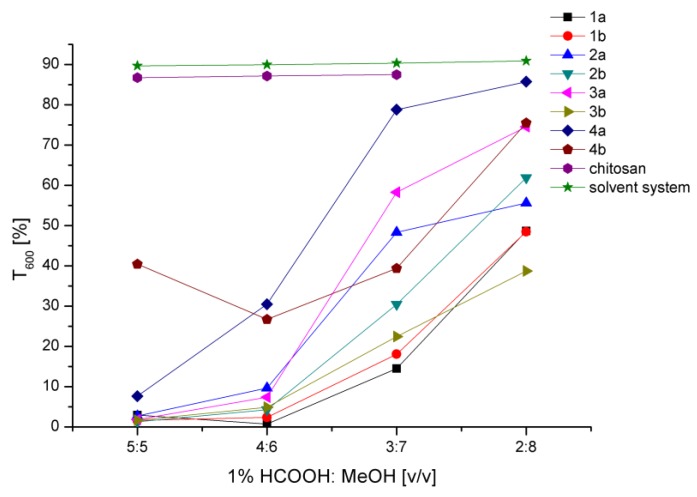
Transparency of chitosan derivatives solutions at different volume ratios of 1 wt% formic acid and methanol.

Solubility study was performed by transmittance spectra analysis (UV-Vis) at the selected wavelength and analyzed in terms of the transparency of the solutions—which is indicative of macromolecules’ conformation [37,38,39,40]. The transparency results of chitosan and derivative solutions with different 1 wt% formic acid/methanol volume ratios are presented in Figure 6. For chitosan solution, the addition of methanol did not change transparency up to the 3:7 (*v*/*v*) ratio. Due to the chitosan gel-like structure observed at 2:8 (*v*/*v*) ratio, the transparency measurement for that system could not be performed. Regarding the derivatives, the addition of methanol to the acidic aqueous solution significantly improved their transparency. Thus, changing the solvent character to make it less aqueous allowed the hydrophobic fatty acid chains to interact with the solvent mixture, enhancing the solubility of the amphiphilic macromolecules which are non-soluble in the purely aqueous solution. The derivative 4b exhibited a high degree of solubility even at a 5:5 (*v*/*v*) ratio, since it is characterized by the lowest substitution degree and thus its solution behavior is dominated by chitosan.

The observed differences in the transparency of the systems were significant, which indicates that modification of chitosan chains with the long fatty acids’ molecules changes the behavior of the macromolecules in solution. The presence of fatty acid in the derivatives disturbs the polyelectrolyte character of chitosan chains forcing the amphiphilic macromolecules to achieve a new spatial configuration, and what is more, hinders the hydrogen bonds occurring between chitosan chains. This situation is beneficial from a practical application point of view of the derivatives, as it leads to the appearance of hydrophobic interactions responsible for the self-assembly properties.

**Figure 7:**
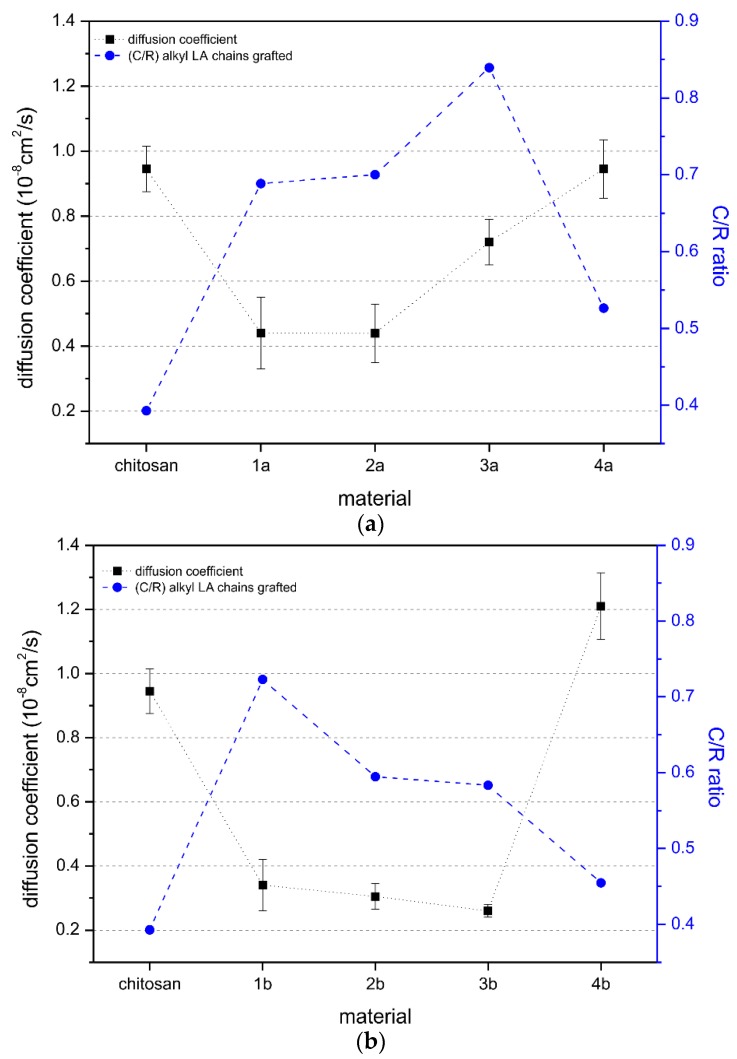
Diffusion coefficient vs. relative amount of grafted linoleic acid for (**a**) the EDC and (**b**) EDC/NHS coupling system.

To evaluate the diffusion behavior of macromolecules, the dynamic light scattering technique was used, based on the cumulants analysis method. This technique provides the mean value of the diffusion coefficient of a macromolecule, assuming Gaussian-like distribution around the mean value. Figure 7 presents the values of the diffusion coefficient for the chitosan and the obtained derivatives, with EDC (Figure 7a) and EDC/NHS (Figure 7b) coupling systems, relative to the number of alkyl chains grafted. Polymer concentration for the analysis was set at 1 mg/mL in 2:8 (*v*/*v*) 1 wt% formic acid/methanol solution.

For the chitosan, the translational diffusion coefficient was found to be 0.945 μm^2^/s, whereas in 1 wt% acetic acid solution, the diffusion coefficient was 1.27µm^2^/s (data not included in the graph). These results confirmed the previous observation that solvent strongly influences the spatial orientation of macromolecules by changing their hydrodynamic radius, which arises from different polymer–polymer and polymer–solvent interactions. It is well established that chitosan can behave like a highly rigid [41,42] or semi-flexible polymer [6,43], depending on the ionic strength of the solution. From a general viewpoint, we have observed that with regards to the measured diffusion coefficient values, chitosan derivatives behave differently than unmodified chitosan in solution. For the derivatives obtained only with EDC, an increasing amount of grafted linoleic acid (derivatives 1a–3a) raised the diffusion coefficient, but the values were still significantly lower compared to the ones for unmodified chitosan (Figure 7a). These results suggest that the spatial conformation in the selected solvent system (1 wt% formic acid in water/methanol, 2:8 *v*/*v*) for the obtained derivatives, restricts the Brownian motions in solution, due to the long alkyl chains grafted on chitosan macromolecules. Only for the derivative 4a, the amount of grafted linoleic acid was not sufficient to cause a/the different orientation of macromolecules in solution and therefore, its diffusion coefficient was comparable to the value obtained for unmodified chitosan. We could simply assume that the observed lower diffusion coefficients resulted in a larger hydrodynamic radius for the derivatives, which is consistent with the Stokes–Einstein equation for the diffusion coefficient of particles undergoing Brownian motion in a quiescent fluid at the uniform temperature [44].

Philippova et al. [45] reported that for hydrophobically modified chitosan (HCM), the addition of alcohol to the aqueous solution changed the self-assembly behavior of HCM macromolecules, since the presence of alcohol affected the surface tension of the solution, diminishing the hydrophobic interactions and hence, the self-assembly forces. However, contrary to our results, in an alcoholic aqueous solution, the authors observed similar behavior of HCM to chitosan. Only 4a derivative, with the lowest fatty acid grafted content among of the EDC obtained derivatives, follows a similar trend to Philippova (i.e., a diffusion coefficient value similar to chitosan). The distinctive behavior of the rest of the derivatives is likely related to the higher fatty acid level. This fact is consistent with the behavior of the 4b derivative, as it has the lowest fatty acid grafted content (lower than 4a), and exhibits an even higher diffusion coefficient than unmodified chitosan. For the rest of the derivatives, there is a general trend of the diffusion coefficient to decrease almost proportionally to the amount of the LA grafted. Therefore, it seems that there is a minimum LA moiety graft needed to alter the spatial conformation and hydrodynamic behavior of macromolecules in solution.

## 3. Materials and Methods

### 3.1. General Information

Chitosan ChitoClear^®^ 43000—hqg10 from Primex ehf Iceland Company (deacetylation degree ~83%, determined by the ^1^H NMR); linoleic acid (LA) (≥99%) 1-ethyl-3-(3-dimethylamino–propyl)carbodiimide hydrochloride (EDC), *N*–hydroxysuccinimide (NHS), acetic acid conc. (AA), hydrochloric acid conc. (HCl), formic acid conc. (FA) and methanol (MeOH) were purchased from Sigma Aldrich Co., Ltd. and used without further purification; anhydrous sodium acetate, deuterated formic acid, deuterated methanol, deuterated water, were purchased from Acros Organics. Pyridine (Py) and chloroform (CHCl_3_) were purchased from POCh, Poland.

### 3.2. Synthesis of Chitosan Derivatives

The synthesis of chitosan derivatives was conducted as a two-step process. In the first step, linoleic acid (LA, 0.214 g, 0.89 mmol, 30 wt%) was dissolved in 15 mL of MeOH and the proper amount of EDC was added (0.17 g, 0.89 mmol, 1 eq. or 0.34 g, 1.78 mmol, 2 eq.). The activation step was conducted for 20 min at room temperature. After this time, NHS was added (0.10 g, 0.89 mmol or 0.20 g, 1.78 mmol, equimolar to EDC; derivative: 1b, 2b, 3b, 4b) and stirred for 10 min. In the second step, the solution was dropwise added to the prior prepared chitosan solution and the reaction was continued for 24 h. Chitosan (0.5 g) solution was prepared using three different reaction media (total volume: 50 mL): (I) 1 wt% acetic acid (final pH= 4.7), (II) 1 wt% AA buffered to pH = 4.0 using HCl (1 M) and (III) water buffered to pH = 4.0 using HCl (1M). To each solution, 15 mL of MeOH was added and stirred for 24 h. After that time, the reaction mixture was dialyzed against water for 48 h at 25 °C (SPECTRA/POR 3, MWCO: 3.5 kDa) and then freeze-dried (freeze dryer, Christ, Alpha 1-2) for 48 h. The final product obtained was a yellowish solid in a sponge-like form.

### 3.3. Solubility Test

Visual evaluation and UV-Vis spectroscopy (Jasco, V-630, Tokyo, Japan) measurements were performed to determine the solubility of the derivatives. Different solvent systems—AA, HCl, FA, MeOH, DMSO, Py, and CHCl_3_—were examined at the derivative concentration of 10 mg/mL (Supporting Information, SI, Table 1). Among all tested solvent mixtures, 1 wt% formic acid and MeOH were selected and further tested to determine the volume ratio of those components leading to the highest transparency of the solution. The transmittance was recorded in a micro-PS cell with an optical path length of 1 cm at the wavelength of 600 nm.

### 3.4. Fourier Transform Infrared Spectroscopy

The chemical structure of chitosan derivatives was assessed by Fourier transform infrared-attenuated total reflection (FT-IR ATR) spectroscopy on a Bruker ALPHA spectrometer. Prior to the analysis, all samples were dried at 40 °C in a vacuum for 24 h. For each sample, 32 scans with 2 cm^−1^ resolution were averaged across the spectral range of 4000–400 cm^−1^.

The substitution degree of the chitosan derivatives was analyzed by comparing the absorption intensities of the characteristic bands as previously described [34]. The band of asymmetric bridge oxygen stretching at 1150 cm^−1^ was chosen as a reference band (R). The characteristic probe bands included amide (A, ν_max_ ≈ 1640 cm^−1^), ester (B, ν_max_ ≈ 1739 cm^−1^) and alkyl (C, ν_max_ ≈ 2855 cm^−1^) functional groups.

### 3.5. Nuclear Magnetic Resonance Spectroscopy

^1^H and ^13^C Nuclear Magnetic Resonance spectroscopic measurements were performed on a Bruker DPX 400 Avance III HD spectrometer, operating at 400.2 MHz and 100.6 MHz, respectively. Approximately 5 mg of each chitosan derivative was dissolved in 500 μL of solvent mixture (2/8 *v*/*v*) consisting of 1 wt% formic acid-d4 in deuterium oxide (*v*/*v*) and methanol-d4 (FA:MeOH). Briefly, samples were allowed to dissolve in FA at 60 °C overnight, then MeOH was added and the system was mixed at room temperature until complete dissolution was observed (SI, Table S2). 4,4-dimethyl-4-silapentane-1-sulfonic acid (DSS) was used as an internal chemical shift standard. The 1D diffusion-filtered ^1^H NMR spectra were acquired using a 1D stimulated spin–echo pulse sequence with bipolar gradients and LED (ledbpgp2s1d) [46] for effective suppression of the water and methanol signals while retaining the signals from the analyzed polymer. The relaxation delay was 3.0 s, the diffusion delay Δ was 50–95 ms, the eddy current time was set to 5 ms and the gradient pulse length δ was 1 ms. The gradient strength was set to 95 % (GPNAM = SMSQ10.100). ^13^C DEPT-135 NMR spectra were acquired using an acquisition time of 1.6 s, delay time of 2 s, the pulse width of 10 µs and a total of 8192 scans.

The digital resolution of the 2D matrix was 0.4 Hz/pt. A 2D HMQC ^13^C-^1^H chemical shift correlation spectrum was recorded by using a 1024 × 1024 data matrix size and 64 scans for each t1 value. The digital resolution of the ^13^C axis was 100.6 Hz and that of the ^1^H axis was 400.2 Hz. The transmitter frequency offset O1 was set to 1953.4 Hz (f1 − ^1^H), and relaxation delay to 1.5 s. An SMSQ10.100 gradient file was used, with gradient values selected for the gradient Z: gpz1, 50%; gpz2, 30%, and gpz3, 40.1%.

### 3.6. Dynamic Light Scattering

The translational diffusion coefficient [D, 10^−8^ cm^2^/s] was evaluated by dynamic light scattering (Zetasizer NanoZS, Malvern Instruments Ltd., Malvern, United Kingdom) for the all derivatives’ solutions at a concentration of 1 mg/mL in 2:8 *v*/*v* 1 wt% formic acid/methanol. All measurements were carried out at 25 °C with the backscatter detection system at the angle 173°. The excitation source was a helium–neon vertically polarized laser operating at a wavelength of 633 nm. Before the measurements, the solutions were filtered using a syringe filter (PES membrane of 1 μm pore size).

### 3.7. Gel Filtration Chromatography

The molecular weights (MWs; M_n_ and M_w_) of the chitosan and the derivatives were determined by gel filtration chromatography-high-performance liquid chromatography (GFC-HPLC) (Shimadzu, Japan) equipped with a refractive index (RI) detector (Model 20A, Shimadzu, Japan), a PolySep™-SEC GFC-P Linear column, LC 300 × 7.8 mm, and with a PolySep-GFC-P Guard Column working at 40 °C. The mobile phase, composed of acetate buffer that consisted of 0.3 M acetic acid and 0.2 M sodium acetate (pH = 4), was eluted at 0.4 mL/min speed. The MWs of the chitosan derivatives were calculated through a calibration curve created with pullulan standards (1.0 mg/mL, M_p_ 9600–708000 Da, PSS) and with LabSolution software (Shimadzu, Japan). An internal standard was toluene. Samples were prepared by direct dissolution in acetate buffer (3.0 mg/mL), the same as used for the mobile phase, by using a Heidolph Polymax 1040 laboratory shaker at 37 °C for 48 h. Following this, the measurement samples were filtered with a syringe filter (PES membrane of 0.45 μm pore size). Sample injection volume was 50 μL.

## 4. Conclusions

In this work, eight chitosan derivatives in three different reaction media were obtained by means of an acylation reaction with linoleic acid. Due to the weak acylating properties of carboxylic groups, EDC and EDC/NHS coupling systems were used. The NHS was added in order to increase the stability of the reactive ester and lead to selective *N*-acylation, however, infrared spectra analysis results demonstrated that an acidic environment and, thus, amino groups’ protonation, hindered the amino groups’ accessibility to the reaction. As a consequence, a lower substitution degree was achieved for the derivatives obtained with the EDC/NHS coupling system compared to the ones obtained with EDC. Additionally, the determination of the substitution place in a quantitative way based on ^1^H NMR was demonstrated to be uncertain due to the overlapping of target signals (CH_2_ α, LA signals with respect to an ester or amide group), which was elucidated by the ^1^H-^13^C HMQC experiment. *N*,*O*-Acylated chitosan derivatives’ chemical structures are rather complex and, thus, their analysis should be performed with great care and in a detailed manner (combining FTIR and ^1^H-^13^C NMR analysis), in order to properly establish relationships with their final behavior. Remarkably, variations in the chemical structure of the derivatives significantly changed their behavior in solution, affecting the molecular weight determination, since the recommended standard dissolved in a different solvent system. In this regard, the presence of grafted fatty acid chains into the chitosan, altered its polyelectrolyte character and therefore, its spatial configuration in solution. For the same reason, the diffusion coefficient of the derivatives (despite one with a very low substitution degree) was much lower than the one for unmodified chitosan.

The presented results provided an argument for the importance of intentionally selecting reaction conditions, as they might exhibit key influences on the derivative’s structure and its further properties. Additionally, the study performed highlights the fact that a deep characterization combining different methods is necessary in order to establish a proper relationship between behavior and structure, as the poor characterization of these kinds of polymers and derivatives often leads to confusion and makes comparisons among similar systems difficult to achieve.

## Figures and Tables

**Table 1:** Molecular weights and polydispersity index for chitosan and chitosan derivatives.

Sample	Number-Average Molecular Weight (M_n_) [kDa]	Weight-Average Molecular Weight (M_w)_ [kDa]	Polydispersity Index (M_w_/M_n_)
chitosan	19.9	145.7	7.3
1a	13.2	53.0	4.0
1b	15.1	68.4	4.5
2a	15.1	62.0	4.1
2b	12.0	69.9	5.8
3a	13.6	65.8	4.8
3b	12.8	63.8	4.9
4a	13.2	66.5	5.0
4b	19.5	145.8	7.5

## Supplementary Materials

The following are available online at https://www.mdpi.com/1420-3049/24/17/3047/s1, Table S1. Solubility in different organic systems for the derivative 3a at the concentration 10 mg/mL; Table S2. Solubility in different acids/methanol systems for the derivative 3a at different concentrations; Figure S1. The comparison of 1H NMR spectra of chitosan derivative 2a measured using different water suppression techniques: standard puls program (zg30)—red spectrum, watergate (zggpwg)—green spectrum, and water presaturation (zgpr)—blue spectrum; Figure S2. ^1^H NMR spectrum of chitosan; Figure S3. ^1^H NMR spectrum of chitosan derivative 1a; Figure S4. ^1^H NMR spectrum of chitosan derivative 1b; Figure S5. ^1^H NMR spectrum of chitosan derivative 2a; Figure S6. ^1^H NMR spectrum of chitosan derivative 2b; Figure S7. ^1^H NMR spectrum of chitosan derivative 3a; Figure S8. ^1^H NMR spectrum of chitosan derivative 3b; Figure S9. ^1^H NMR spectrum of chitosan derivative 4a; Figure S10. ^1^H NMR spectrum of chitosan derivative 4b.
