# Supplementary material for: The Importance of Reaction Conditions on the Chemical Structure of N,O-Acylated Chitosan Derivatives
Source: Molecules. 2019 Aug 22;24(17):3047. doi: 10.3390/molecules24173047 (PMC6749269; doi:10.3390/molecules24173047)
Supplement: Supplementary file 1 [file molecules-24-03047-s001.pdf]

Supporting Information

Table S1. Solubility in different organic systems for the derivative 3a at the concentration 10 mg/ml.

No.	1%AA [mL]	MeOH [mL]	$CHCl_3$ [mL]	DMSO [mL]	Pyridyne [mL]	dissolution procedure	conditions	Solution appearance
1.	0.5	0.25	0.25	-	-	all solvents added at once in order: 1%AA, MeOH, $CHCl_3$	a) RT, 3 days<br>b) 60 °C, 24 h	a) turbid<br>b) turbid
2.	0.5	0.17	0.33	-	-	all solvents added at once in order: 1%AA, MeOH, $CHCl_3$	a) RT, 3 days<br>b) 60 °C, 24 h	a) turbid<br>b) turbid
3.	0.5	0.12	0.38	-	-	all solvents added at once in order: 1%AA, MeOH, $CHCl_3$	a) RT, 3 days<br>b) 60 °C, 24 h	a) turbid<br>b) turbid
4.	0.5	0.4	0.1	-	-	all solvents added at once in order: 1%AA, MeOH, $CHCl_3$	a) RT, 3 days<br>b) 60 °C, 24 h	a) turbid<br>b) turbid
5.	0.5	0.1	0.4	-	-	all solvents added at once in order: 1%AA, MeOH, $CHCl_3$	a) RT, 3 days<br>b) 60 °C, 24 h	a) turbid<br>b) turbid
6.	0.5	0.5	-	-	0.1	a) all solvents added at once in order: 1%AA, MeOH, $CHCl_3$<br>b) continuation of dissolution at selected temperature<br>c) pyridine addition<br>d)continuation of dissolution at selected temperature	a) RT, 3 days<br>b) 60 °C, 24 h<br>c) RT<br>d) 40 °C, short mixing	a) turbid<br>b) less turbid<br>c) partial dissolution<br>d) complete dissolution
7.	0.5	-	-	0.5	0.1	a) all solvents added at once in order: 1%AA, DMSO<br>b) continuation of dissolution at selected temperature<br>c) pyridine addition<br>d) continuation of dissolution at selected temperature	a) RT, 3 days<br>b) 60 °C, 24 h<br>c) RT<br>d) 40°C, short mixing	a) turbid<br>b) less turbid<br>c) partial dissolution<br>d) partial dissolution
8.	-	0.5	-	0.5		a) all solvents added at once in order: MeOH, DMSO<br>b) continuation of dissolution at selected temperature	a) RT, 3 days<br>b) 60 °C, 24 h	a) turbid<br>b) gelation
9.	0.4	0.3	-	0.3	0.1	a) all solvents added at once in order: 1%AA, MeOH, DMSO<br>b) continuation of dissolution at selected temperature<br>c) pyridine addition<br>d)continuation of dissolution at selected temperature	a) RT, 3 days<br>b) 60 °C, 24 h<br>c) RT<br>d) 40 °C, short mixing	a) turbid<br>b) less turbid<br>c) partial dissolution<br>d) partial dissolution
10.	0.2	0.4	-	0.4	-	a) all solvents added at once in order: 1%AA, MeOH, DMSO<br>b) continuation of dissolution at selected temperature	a) RT, 3 days<br>b) 60 °C, 24 h	a) turbid<br>b) gelation
11.	0.3	0.7	-	-	0.1	a) all solvents added at once in order: 1%AA, MeOH<br>b) continuation of dissolution at selected temperature<br>c) pyridine addition	a) RT, 1 day<br>b) 60 °C, 24 h<br>c) RT	a) gelation<br>b) partial dissolution<br>c) complete dissolution
12.	0.7	0.3	-	-	0.1	a) all solvents added at once in order: 1%AA, MeOH<br>b) continuation of dissolution at selected temperature<br>c) pyridine addition<br>d) continuation of dissolution at selected temperature	a) RT, 1 day<br>b) 60 °C, 24 h<br>c) RT, 24 h<br>d) 60 °C, 4-5 h	a) gelation<br>b) gelation<br>c) turbid<br>d) turbid
13.	0.3	-	0.7	-	0.1	a) all solvents added at once in order: 1%AA, DMSO<br>b) continuation of dissolution at selected temperature<br>c) pyridine addition<br>d) continuation of dissolution at selected temperature	a) RT, 1 day<br>b) 60 °C, 24 h<br>c) RT, 24 h<br>d) 60 °C, 4-5 h	a) gelation<br>b) gelation<br>c) transparent gel<br>d) transparent gel
14.	0.7	-	0.3	-	0.1	a) all solvents added at once in order: 1%AA, DMSO	a) RT, 1 day<br>b) 60 °C, 24 h	a) gelation<br>b) no dissolution

						b) continuation of dissolution at selected temperature<br>c) pyridine addition<br>d) continuation of dissolution at selected temperature	c) RT, 24 h<br>d) 60 °C, 4-5 h	c) turbid<br>d) partial dissolution
15.	0.5	-	0.4	0.1	-	a) all solvents added at once in order: 1%AA, pyridine<br>b) continuation of dissolution at selected temperature	a) RT, 1 day<br>b) 60 °C, 24 h	a) gelation<br>b) partial dissolution
16.	0.5	0.1	-	-	0.5	a) all solvents added at once in order: 1%AA, pyridine<br>b) continuation of dissolution at selected temperature<br>c) MeOH addition	a) RT, 1 day<br>b) 60 °C, 24 h<br>c) 60 °C, 24 h	a) no dissolution<br>b) partial dissolution<br>c) partial dissolution
17.	-	-	-	-	1		a) RT, 1 day<br>b) 60 °C, 24 h	a) no dissolution<br>b) gelation
18.	-	0.5	-	-	0.5	a) all solvents added at once in order: MeOH, pyridine<br>b) continuation of dissolution at selected temperature	a) RT, 1 day<br>b) 60 °C, 24 h	a) no dissolution<br>b) gelation
22.	0.2	0.8	-	-	-	a) dispersion in acid<br>b) MeOH addition	a) 60 °C, overnight<br>b) 60 °C, 4-5 h	a, b) partial dissolution
23.	0.1	0.9	-	-	-	a) dispersion in acid<br>b) MeOH addition	a) 60 °C, overnight<br>b) 60 °C, 4-5 h	a, b) partial dissolution
24.	0.3	0.7	-		0.05	a) dispersion in acid<br>b) MeOH addition<br>c) pyridine addition	a) 60 °C, overnight<br>b) 60 °C, 4-5 h<br>c) 60 °C	a, b) slightly turbid<br>c) complete dissolution

Table S2. Solubility in different acids/methanol systems for the derivative 3a at different concentrations.

No.	concentration [g]	1% HCl [mL]	1% FA [mL]	MeOH [mL]	dissolution procedure	conditions	Solution appearance
1.	10 mg/mL	0.3	-	0.7	a) dispersion in acid<br>b) MeOH addition<br>c) continuation of dissolution	a) 60 °C, overnight<br>b) rt,<br>c) 60 °C, 1h	a) turbid<br>b) partial dissolution<br>c) complete dissolution
2.		-	0.3	0.7	a) dispersion in acid<br>b) MeOH addition<br>c) continuation of dissolution	a) 60 °C, overnight<br>b) rt,<br>c) 60 °C, 1h	a) turbid<br>b) partial dissolution<br>c) complete dissolution
3.		1	-	0.1	a) dispersion in acid<br>b) MeOH addition<br>c) continuation of dissolution	a) 60 °C, overnight<br>b) rt<br>c) 60 °C, 1 h	a) turbid<br>b) turbid<br>c) turbid
4.		-	0.2	0.8	a) dispersion in acid<br>b) MeOH addition<br>c) continuation of dissolution	a) 60 °C, overnight<br>b) rt,<br>c) 60 °C, 1h	a) turbid<br>b) partial dissolution<br>c) complete dissolution
5.		-	0.3	0.8	a) dispersion in acid<br>b) MeOH addition<br>c) continuation of dissolution	a) 60 °C, overnight<br>b) rt,<br>c) 60 °C, 1h	a) turbid<br>b) complete dissolution
6.		-	0.2	0.9	a) dispersion in acid<br>b) MeOH addition<br>c) continuation of dissolution	a) 60 °C, overnight<br>b) rt,<br>c) 60 °C, 1h	a) turbid<br>b) partial dissolution<br>c) complete dissolution
7.		-	0.1	0.9 + 0.1	a) dispersion in acid<br>b) MeOH addition	a) 60 °C, overnight<br>b) 60 °C, 1h	a) turbid<br>b) complete dissolution
8.	20 mg/mL	-	0.1	0.9 + 0.1	a) dispersion in acid<br>b) MeOH addition<br>c) MeOH addition 0,1 ml	a) 60 °C, overnight<br>b) 60 °C, 1h<br>c) 60 °C, 5 days	a) turbid<br>b) gelation<br>c) partial dissolution
9.	30 mg/mL	-	0.1	0.9 + 0.1	a) dispersion in acid<br>b) MeOH addition<br>c) MeOH addition 0,1 ml	a) 60 °C, overnight<br>b) 60 °C, 1h<br>c) 60 °C, 5 days	a) turbid<br>b) gelation<br>c) no changes- gelation

10.	40 mg/mL	-	0.1	0.9 + 0.1	a) dispersion in acid<br>b) MeOH addition<br>c) MeOH addition 0,1 ml	a) 60 °C, overnight<br>b) 60 °C, 1h<br>c) 60 °C, 5 days	a) turbid<br>b) gelation<br>c) no changes- gelation
11.	50 mg/mL	-	0.1	0.9 + 0.1	a) dispersion in acid<br>b) MeOH addition<br>c) MeOH addition 0,1 ml	a) 60 °C, overnight<br>b) 60 °C, 1h<br>c) 60 °C, 5 days	a) turbid<br>b) gelation<br>c) no changes- gelation
12.	20 mg/mL	-	0.2	0.8	a) dispersion in acid<br>b) MeOH addition	a) 60 °C, overnight<br>b) 60 °C, 5 days	a) turbid<br>b) gelation
13.	20 mg/mL	-	0.3	0.7	a) dispersion in acid<br>b) MeOH addition	a) 60 °C, overnight<br>b) 60 °C, 5 days	a) turbid<br>b) gelation

Solubility tests were performed in terms to define the proper solvents systems, which indicates the derivatives hydrodynamic properties and is crucial for good quality 1H NMR measurements. High transparency of the solution, is indicative of the solubility quality, and is sufficient for the NMR spectra clearness, taking into account also derivative and solvent signals separation (for correct integrals evaluation).

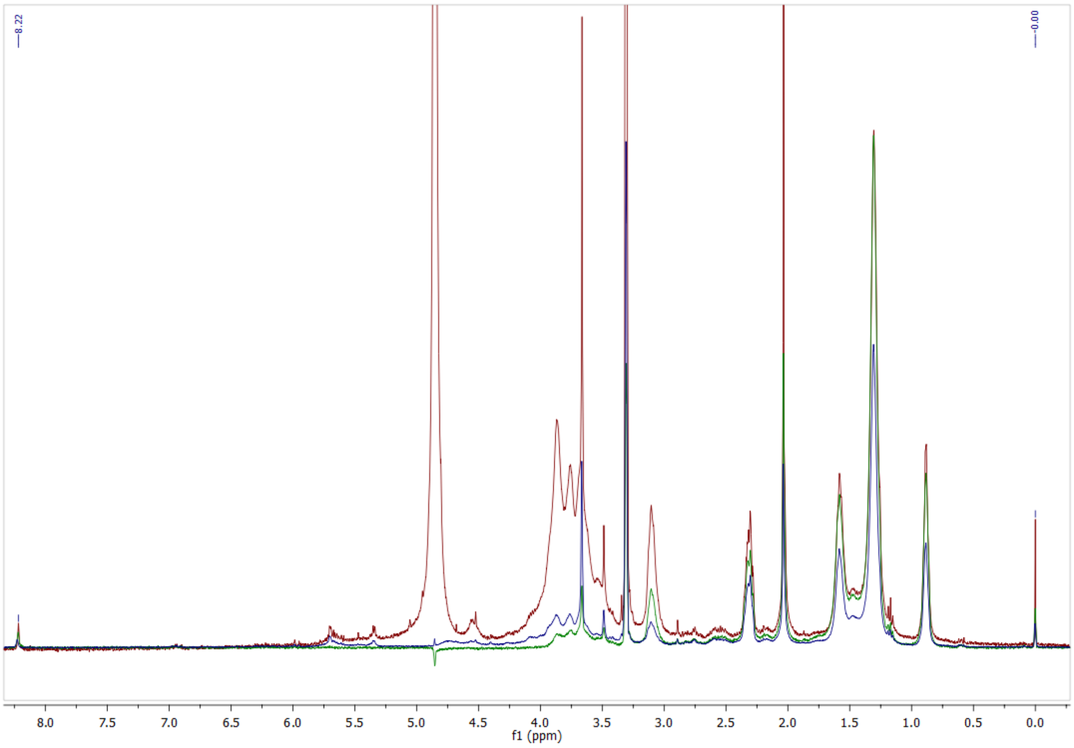

Figure S1. The comparison of ^{1}*H NMR spectra of chitosan derivative* ***2a*** *measured using different water suppression techniques: standard puls program (zg30) – red spectrum, watergate (zggpwg) – green spectrum, and water presaturation (zgpr) – blue spectrum.*

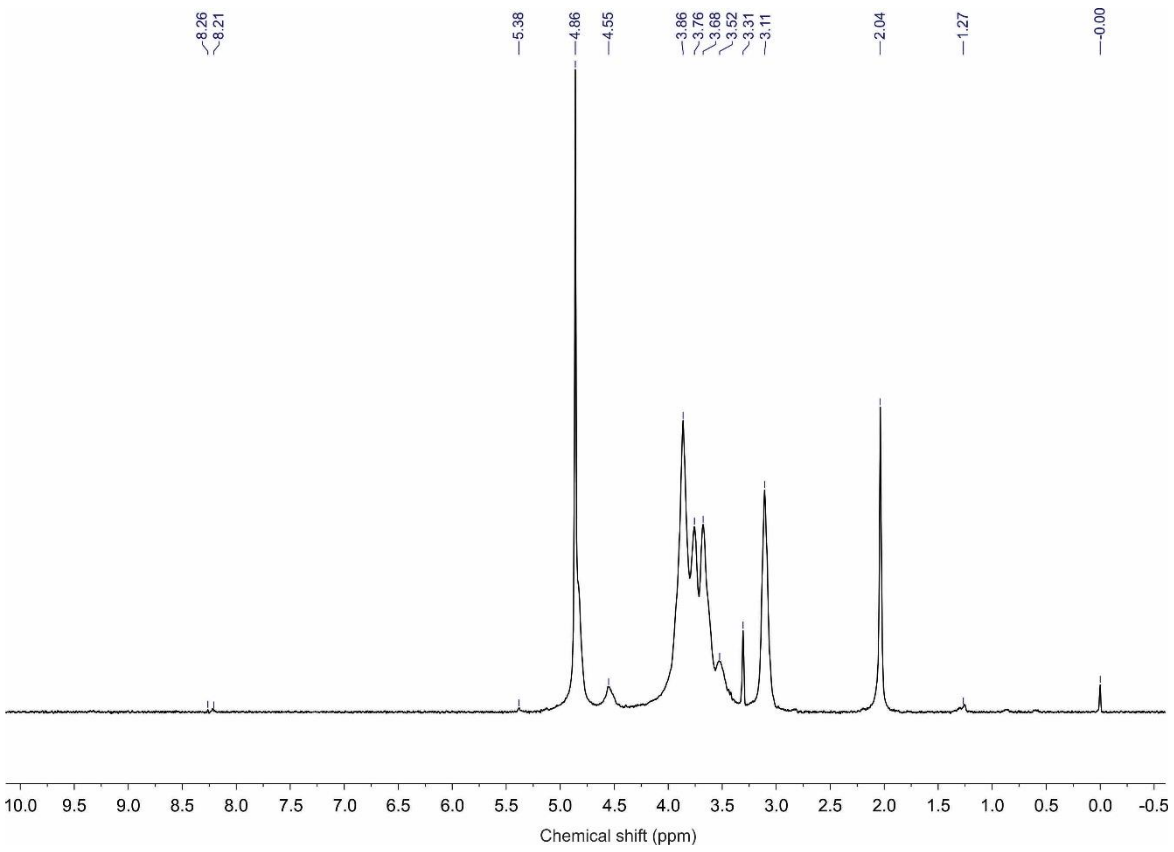

Figure S2. *^{1}H NMR spectrum of chitosan*

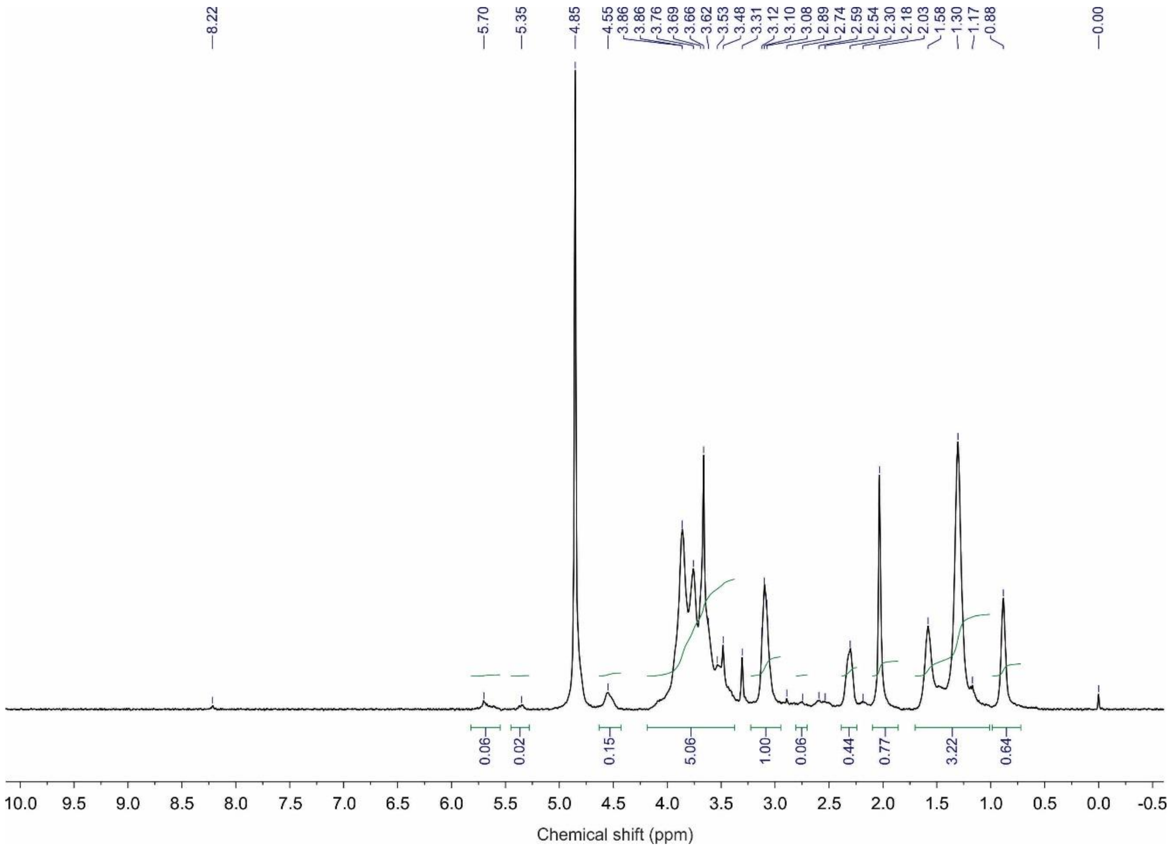

Figure S3. *^{1}H NMR spectrum of chitosan derivative* ***1a***

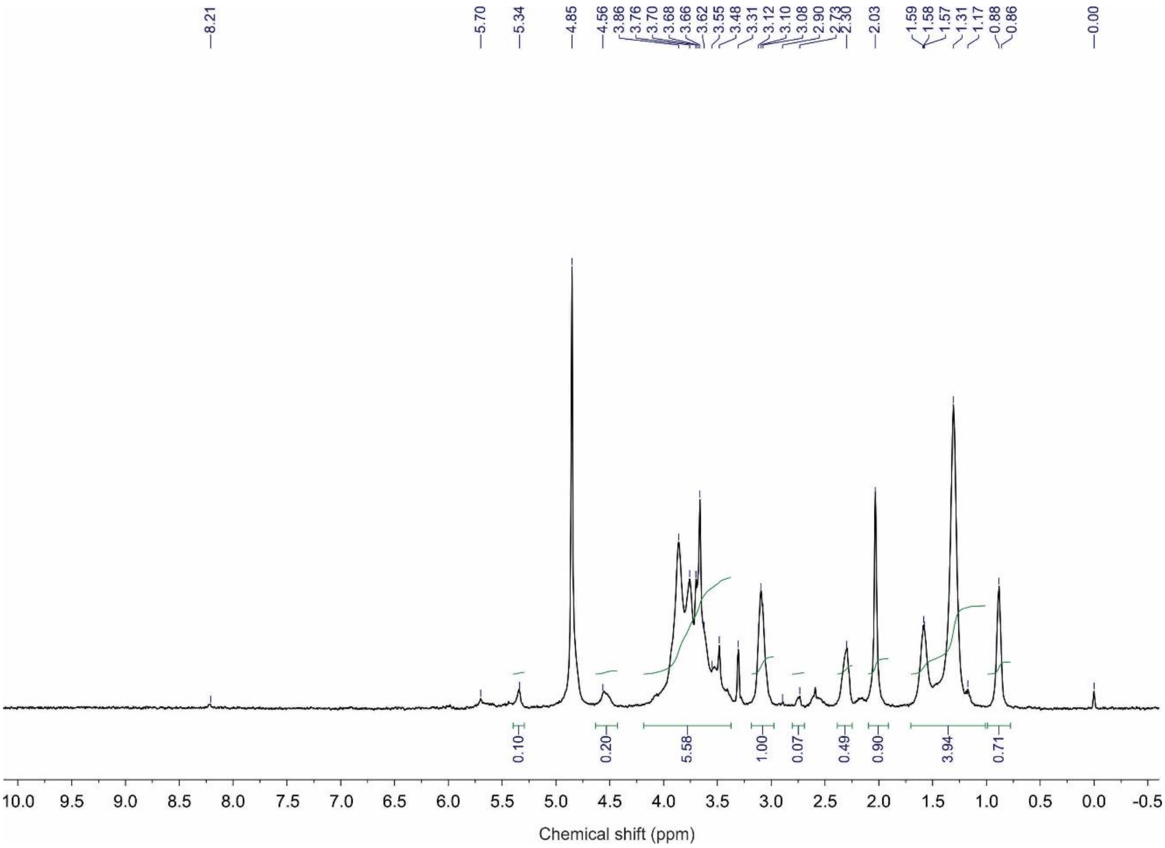

Figure S4. *^{1}H NMR spectrum of chitosan derivative* ***1b***

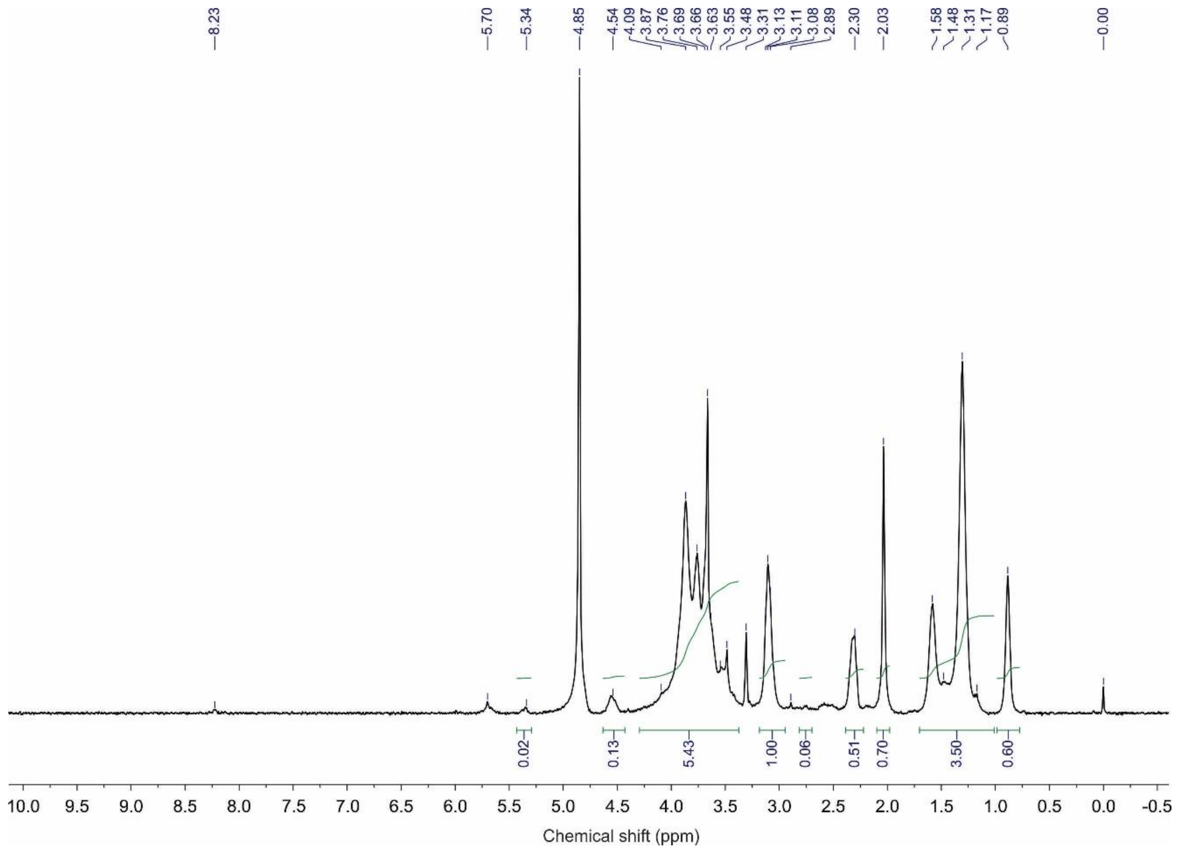

Figure S5. *^{1}H NMR spectrum of chitosan derivative* ***2a***

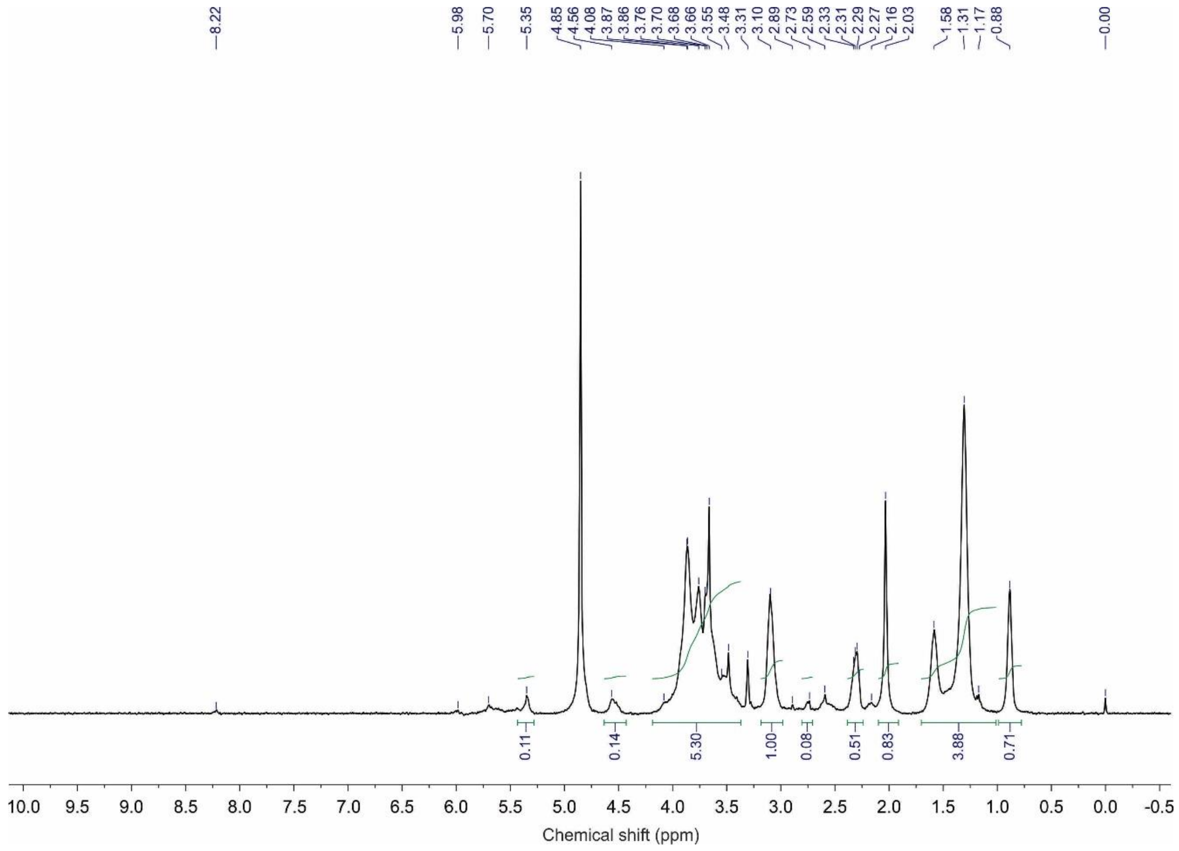

Figure S6. *^{1}H NMR spectrum of chitosan derivative* ***2b***

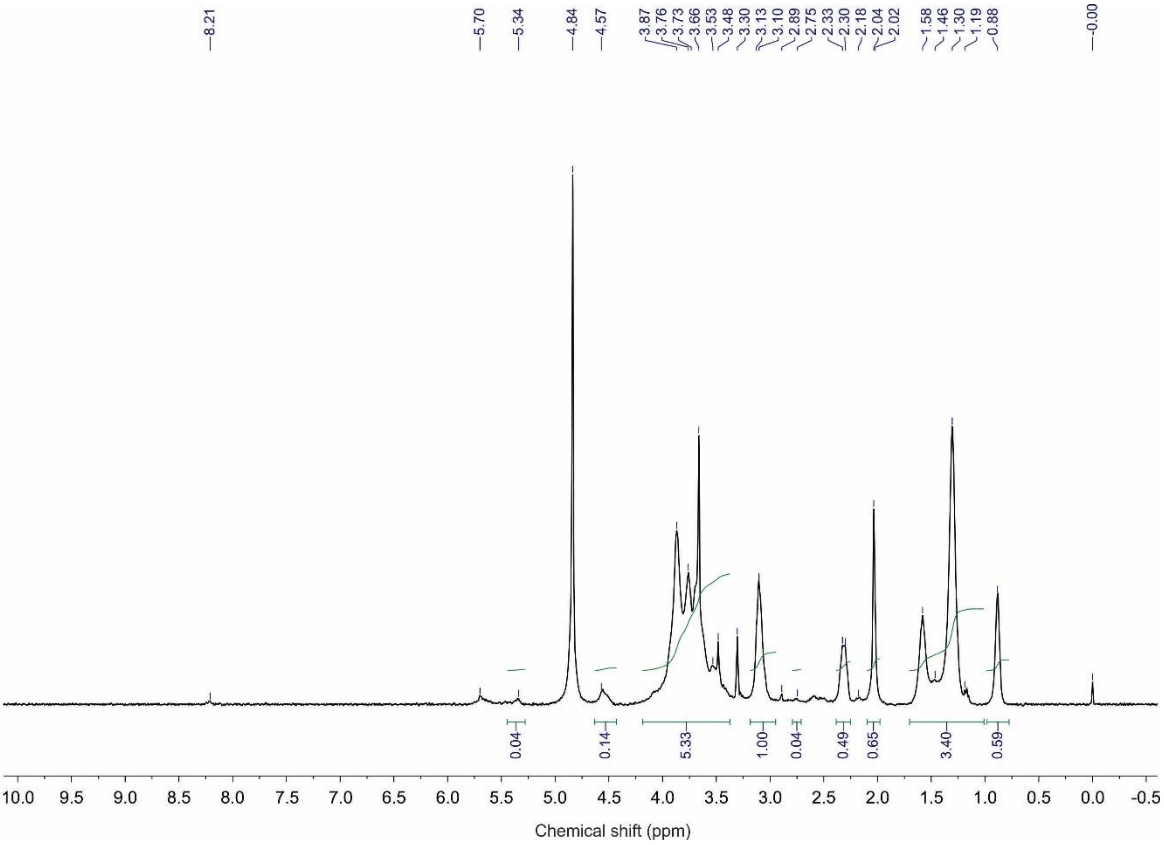

Figure S7. *^{1}H NMR spectrum of chitosan derivative* ***3a***

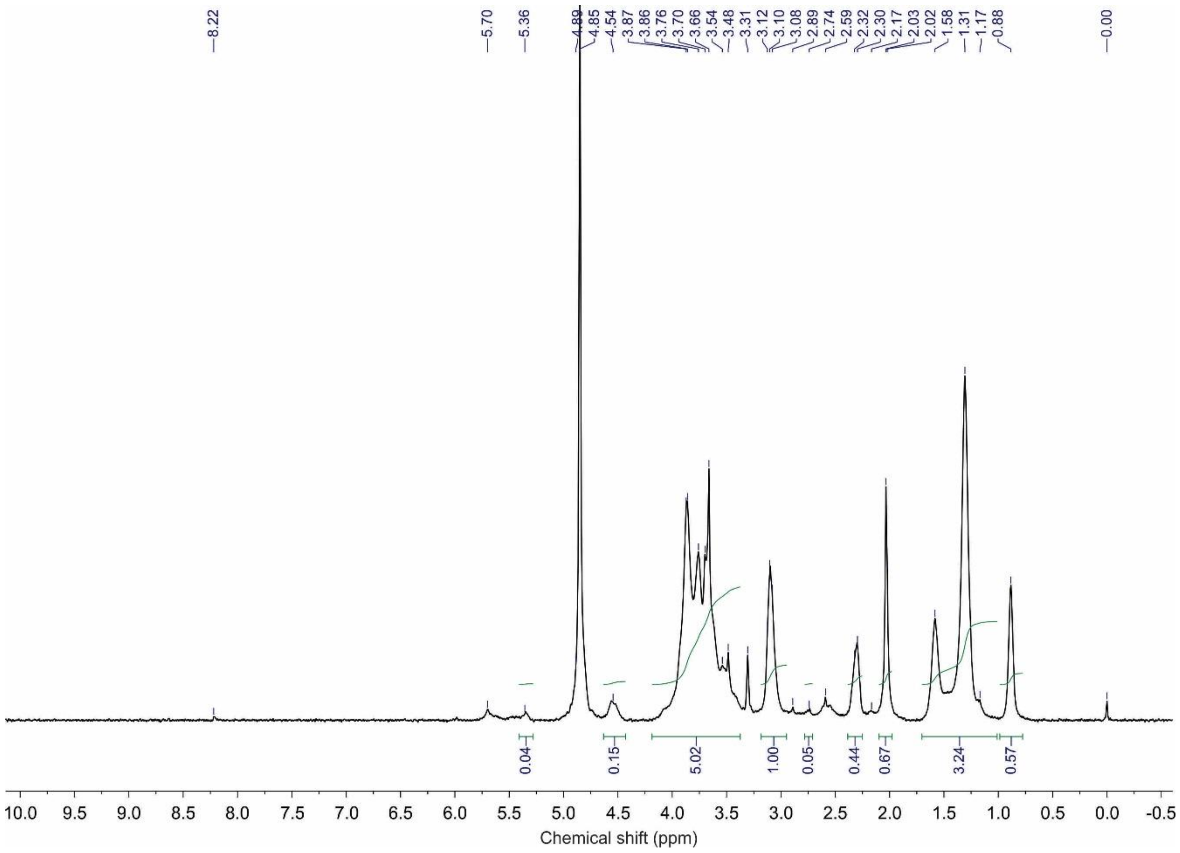

Figure S8. *¹H NMR spectrum of chitosan derivative* ***3b***

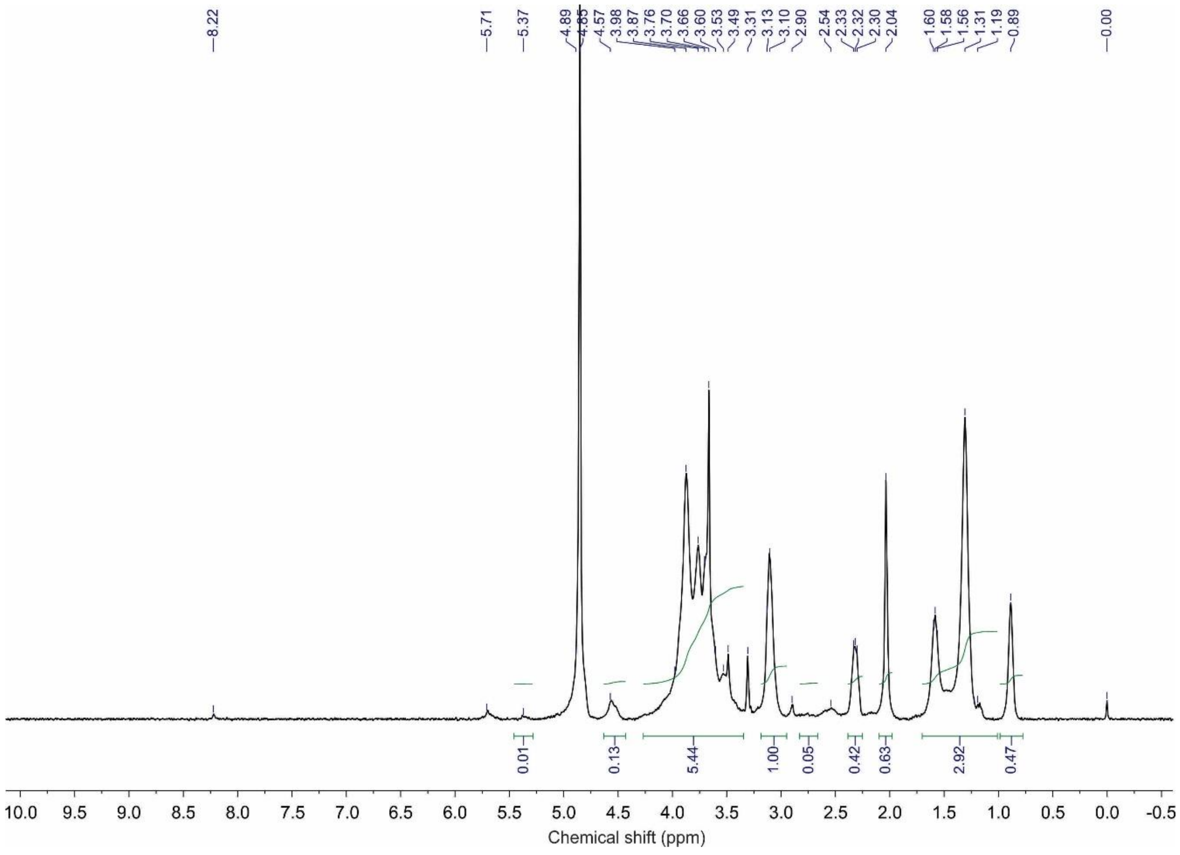

Figure S9. *^{1}H NMR spectrum of chitosan derivative* ***4a***

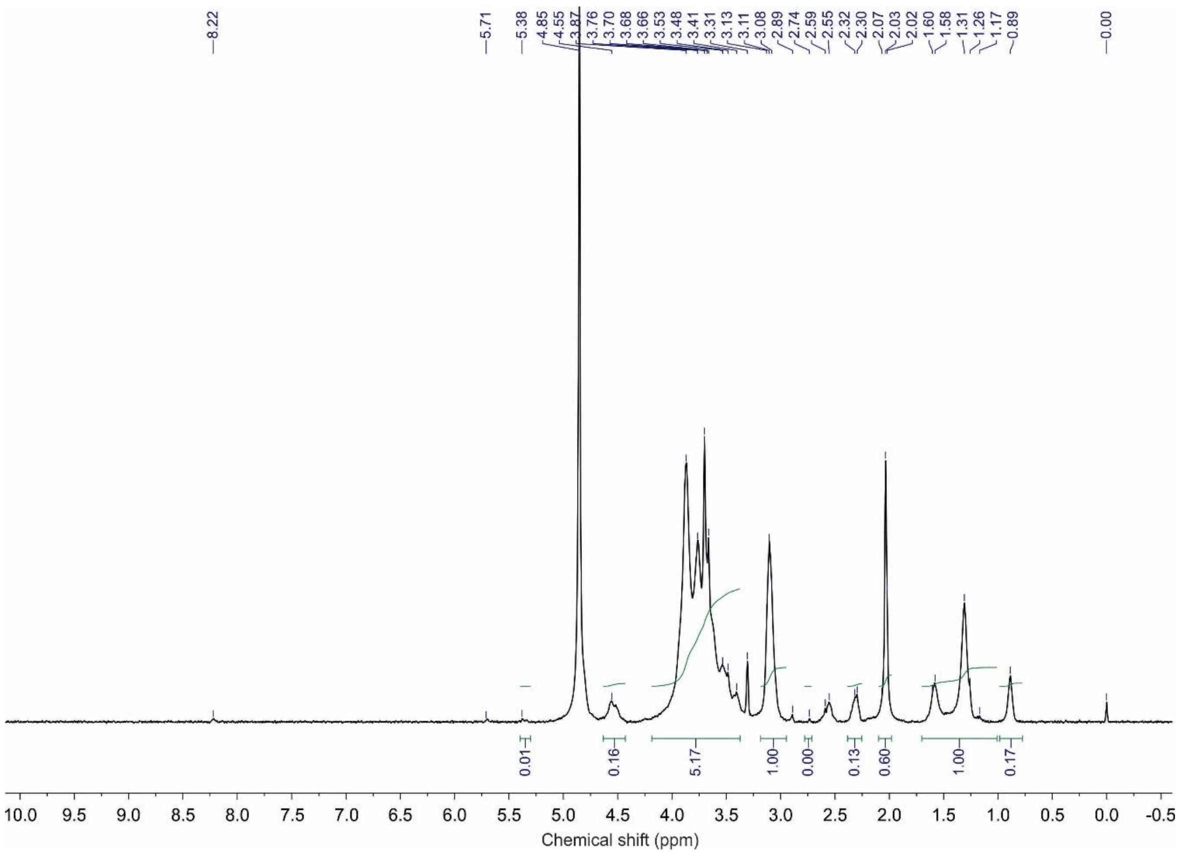

Figure S10. *^{1}H NMR spectrum of chitosan derivative* ***4b***
